# Outpatient Management of Epistaxis During COVID-19 to Reduce Inpatient Stay: A Quality Improvement Project

**DOI:** 10.7759/cureus.30858

**Published:** 2022-10-29

**Authors:** Kaso Ari, Rachael Collins

**Affiliations:** 1 General Surgery, Norfolk and Norwich University Hospital, Norwich, GBR; 2 Otolaryngology, James Paget University Hospital, Great Yarmouth, GBR

**Keywords:** covid-19, service improvement, ent surgery, clincal audit, epistaxis management

## Abstract

Introduction

In March 2020, new guidelines allowed patients with epistaxis to be discharged home with nasal packs in situ to reduce the risk of inpatient coronavirus disease 2019 (COVID-19) transmission rates. Our objective is to review how successful these new guidelines have been and whether they could be safely maintained in future practice.

Methods

This was a retrospective data analysis at a local tertiary ENT referral hospital. The study group consisted of patients admitted with epistaxis over one year. The “Pack and Home” criteria pathway was implemented. We reviewed this pathway six months pre- (loop 1) and six months post- (loop 2) introduction. Primary outcome measures included compliance with the “Pack and Home” criteria and length of inpatient admissions.

Results

A total of 131 patients required nasal packing, with 72 patients (55%) in loop 1 and 59 patients (45%) in loop 2. In loop 1, all 72 patients (100%) were admitted for inpatient care. However, in loop 2, 21 patients (36%) were discharged home with nasal packs in situ and 59 patients (64%) were admitted. Of those discharged, two patients were represented after 48 hours with rebleeding. The average total length of inpatient stay in loop 1 was significantly higher at 45.7 hours and 29.6 hours in loop 2 (p<0.05). All discharged patients attended their outpatient appointment in under three days.

Conclusion

The "Pack and Home" criteria can successfully identify patients who are suited for an outpatient management pathway. This could reduce surgical inpatient stay and the way we manage epistaxis.

## Introduction

Epistaxis is a common presentation to ear, nose, and throat (ENT) departments across the United Kingdom with the majority of requiring hospital admission despite most patients requiring no further intervention other than simple nasal packing [1,2]. Due to its involvement with the upper respiratory tract and being described as an aerosol-generating procedure, the management of epistaxis posed an increased risk of the spread of SARS-CoV-2 to staff and patients [3]. In March 2020, new guidelines were altered to enable certain patients to be discharged home with nasal packs in situ to reduce the risk of coronavirus disease 2019 (COVID-19) inpatient transmission [4]. The literature has highlighted the implications around safe discharge, reduced hospital stay, and readmission rates for patients with epistaxis [5,6].

A criterion was created to help identify patients that would be suitable for outpatient management. This included their social circumstances, stability of patients, observation parameters, and past medical history. Correct adherence to the new guideline could lead to reduced admission of epistaxis patients and reduced hospital stay [7]. The objective of this audit study was to review how successful the new “Pack and Home” criteria pathway has been during the COVID-19 pandemic and whether they could be safely maintained in future practice use.

This article will be presented as a poster abstract at the ASiT Future Surgery 2022 meeting. In addition, this article was published as a preprint on Authorea on September 23, 2022.

## Materials and methods

**Figure 1:**
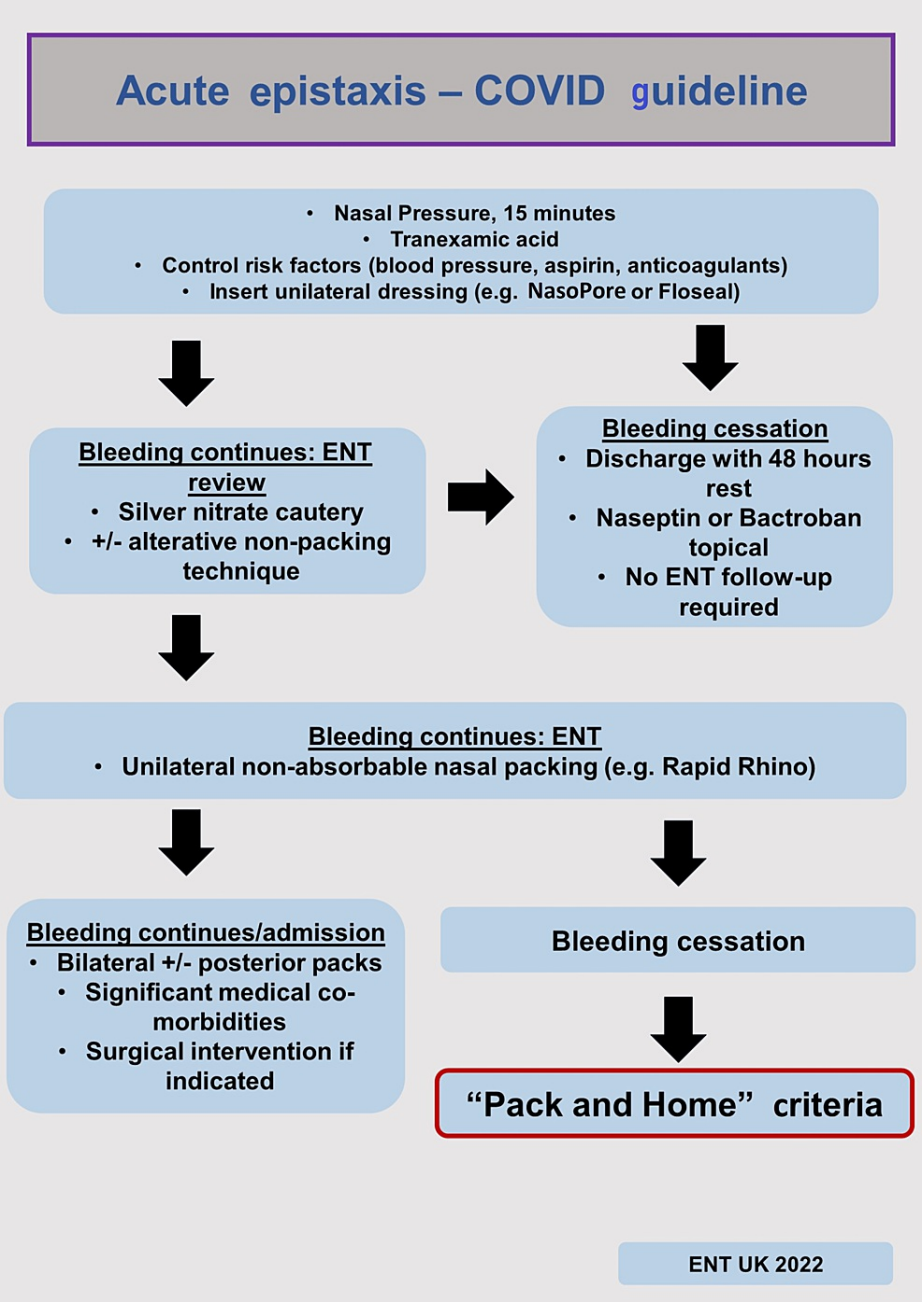
Coronavirus disease 2019 (COVID-19) acute epistaxis management. The figure is adapted from Radulesco et al. [8].

**Figure 2:**
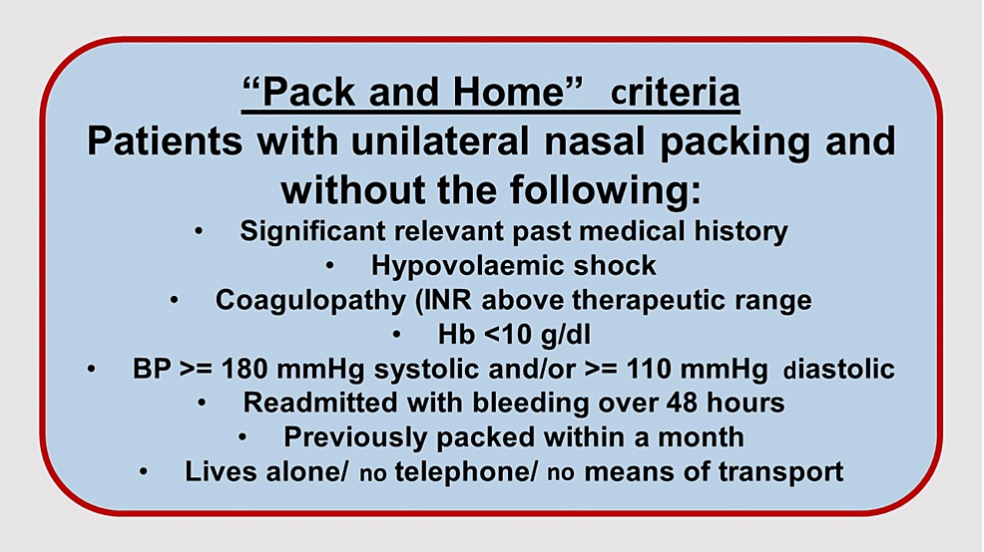
"Pack and Home" criteria.

This was a retrospective data analysis of pre- and post-implementation of the new guidelines which were introduced in March 2020. The “Acute Epistaxis COVID guideline” (Figure 1) with “Pack and Home” criteria (Figure 2) were distributed among accidents and emergency (A&E) staff and ENT clinicians within the hospital in the form of posters around the department, email notifications, and access to hospital guidelines on the intranet.

Firstly, we reviewed the pathway prior to the new guidelines over six months (loop 1 - September 2019 to March 2020) and then reviewed six months post-new guidelines (loop 2 - March 2020 to September 2020). The study group consisted of all patients, aged 18 years and older, admitted to A&E with epistaxis requiring nasal packing as a form of management between September 2019 and September 2020. Primary outcome measures included compliance with "Pack and Home" criteria and length of inpatient stay. Secondary outcome measures included rebleeding post-pack removal, representation with bleeding within 48 hours, and representation within one month.

A descriptive analysis was performed of the baseline clinical characteristics between patients studied in loop 1 and loop 2 of the audit study. Percentages were used for the categorical variables while mean and standard deviations for the continuous variables. The t-test was used to investigate for associations between continuous variables, whereas the chi-squared test of association was used for categorical variables. Non-parametric testing (Mann-Whitney U test) had been used for length of stay. The level of statistical significance was set at p<0.05 and confidence intervals were reported at the 95% level. SPSS version 28 (Armonk, NY: IBM Corp.) was used for statistical analysis.

The terms “Pack and Home” criteria and “outpatient management pathway” are used synonymously in this paper. Patients that were deemed not to be successfully managed on the outpatient pathway were those who did meet the criteria for the “Pack and Home” pathway or were represented with bleeding within 48 hours of discharge.

## Results

Four hundred fourteen patients presented to A&E with epistaxis in loop 1 and 309 patients in loop 2, of which 72 (17.4%) and 59 (19.1%) patients required nasal packing, respectively. This made up the final study population, their clinical characteristics are demonstrated in Table 1. The two loops were not significantly different (p>0.05) with regard to age, sex, and nasal pack types. However, patients in loop 1 were more likely to be on an anticoagulant including direct oral anticoagulants (DOACS), warfarin, and antiplatelets. Patients in loop 1 were more likely to have a past medical history of atrial fibrillation (AF) whereas no difference was demonstrated in the prevalence of other comorbidities between the two groups.

**Table 1:** Baseline information of loop 1 and loop 2 cohort of patients. HTN: hypertension; AF: atrial fibrillation; CVA: cerebrovascular accident; CVD: cardiovascular disease; diabetes M: diabetes mellitus

Demographic		Loop 1	Loop 2	Total	p-Value
Epistaxis presentations, n		414	309	723	-
Packed, n (%)		72 (17.4)	59 (19.1)	131	-
Sex, n (%)	Male	28 (38.9)	28 (47.5)	56 (42.7)	0.324
	Female	44 (61.1)	31 (52.5)	75 (57.3)	
On anticoagulant, n (%)	Yes	38 (52.8)	17 (28.8)	55 (42)	0.006
	No	34 (47.2)	42 (71.2)	76 (58)	
Comorbidities, n (%)	HTN	30 (63.8)	17 (36.2)	47	0.127
	AF	27 (71.1)	11 (28.9)	38	0.018
	CVD	20 (55.6)	16 (44.4)	36	0.933
	Diabetes M	4 (40)	6 (60)	10	0.322
	CVA	10 (66.7)	5 (33.3)	15	0.333

**Figure 3:**
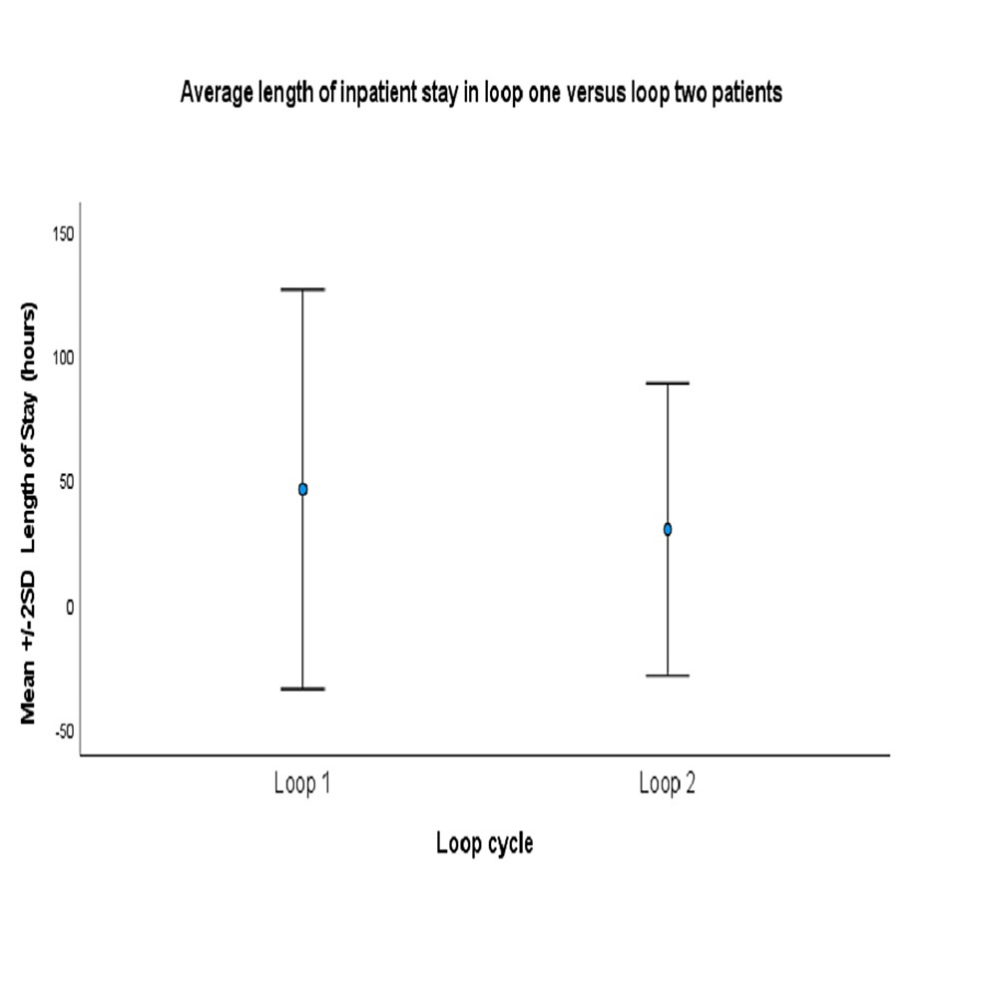
Primary outcome measure - average length of inpatient stay.

Table 2 shows the outcome measures for patients in loop 1 and loop 2 of the audit study. A total of 59 patients required nasal packing in loop 2 of the audit of which 38 patients (64.4%) received inpatient care whereas 21 patients (35.6%) had outpatient care, thus outlining those that had compliance with the criteria. In loop 2 of the audit study, 56 patients (94.9%) were successfully discharged while three patients (5.1%) represented within 48 hours. These three patients were all on the “Pack and Home” pathway. All patients discharged with a nasal pack were reviewed in outpatient clinic in under three days with nearly one-third seen within 24 hours of pack removal and consideration for nasal cautery. No difference was demonstrated for those who had represented with epistaxis within one month between the two study cohorts. Figure 3 demonstrates the average total length of inpatient stay in loop 1 to be significantly higher at 45.7 hours whereas only 29.6 hours in loop 2 (p<0.05).

**Table 2:** Outcome measures of loop 1 cohort versus loop 2 cohort.

Clinical variable		Loop 1	Loop 2	Total	p-Value
Management, n (%)	Inpatient	72 (100)	38 (64.4)	110 (84)	<0.001
	Outpatient	0 (0)	21 (35.6)	21 (16)	
Time to outpatient appointment	1 day	-	14 (66.7)	-	-
	2 days	-	5 (23.8)	-	
	3 days	-	2 (9.5)	-	
Representation within 48 hours, n (%)	Yes	0	3 (5.1)	3 (2.3)	<0.001
	No	72 (100%)	56 (94.9)	128 (97.7)	
Representation within one month, n (%)	Yes	11 (15.3)	9 (15.3)	20 (15.3)	0.997
	No	61 (84.7)	50 (84.7)	111 (84.7)	
Rebled post-pack removal, n (%)	Yes	5 (6.9)	7 (11.9)	12 (9.2)	0.285
	No	65 (90.3)	52 (88.1)	117 (89.3)	
	Unknown	2 (2.8)	0	2 (1.5%)	
Total length of inpatient stay (mean hours±SD)	-	45.7 (±40.1)	29.6 (±29.8)	-	<0.001
	Range	12-264	6-144	-	-

Presentations unsuccessfully sent home with packs

Three patients on the “Pack and Home” criteria re-presented within 48 hours of discharge. Therefore, these patients were not successfully managed on the outpatient pathway successfully due to having a rebleed within 48 hours of discharge requiring return to hospital. Of these three patients, one had a background of ischemic heart disease and valve replacement requiring anticoagulation. One patient had no comorbidities, whereas the other had a history of prostate cancer. Furthermore, two of these patients that returned with bleeding received nasal packing with Merocel® (North Haven, CT: Medtronic), whereas the third patient returned to the hospital complaining of pain from the packing but no actual bleeding.

Rebleeding post-pack removal

Five patients in loop 1 rebled post-pack removal. Of these, three immediately bled, one represented at 12 hours, and one represented at 24 hours. Three of these patients had comorbidities including hypertension and were on anticoagulants. One patient had nasal polyps and the other was fit and well. In loop 2, seven patients had rebled, of which three were on the outpatient pathway and four on the inpatient pathway. Two on the outpatient pathway had no significant comorbidities, one had ischemic heart disease with valve replacement and therefore did not meet the “Pack and Home” criteria. Four inpatient rebleeders had a history of hypertension or were on anticoagulants.

Representation within month

No significant difference was demonstrated for this outcome measure between loop 1 and loop 2 patients (p>0.05). In loop 1, a total of 11 patients had comorbidities including hypertension and/or taking anticoagulants. In loop 2, five patients were healthy whereas four had hypertension and/or were taking anticoagulants.

## Discussion

This audit investigated the compliance rate and safety implications of the new “Pack and Home” criteria guidelines implemented during the COVID-19 pandemic for patients with epistaxis requiring nasal packing. Prior to the pandemic, all with epistaxis were admitted as inpatients, whereas following the introduction of the new guidelines approximately one-third with nasal packs in situ met the criteria and did not require inpatient admission. The new “Pack and Home” criteria resulted in the avoidance of 21 admissions over six months, therefore demonstrating an adequate compliance rate with the new guidelines. Three patients on the new pathway represented within 48 hours, of which in retrospect one did not meet the criteria and should not have been put on the outpatient pathway whereas one represented with pain and the other with bleeding. Thus, with regard to safety implications, only one patient on the “Pack and Home” pathway was represented with bleeding within 48 hours.

The INTEGRATE audit study was able to show that patients can be safely discharged with epistaxis and managed on an outpatient basis [1]. Avoidance of admission has implications towards reduced bed occupancy on surgical wards and reduced opportunities for transmission of hospital-acquired infections. Like our study, the INTEGRATE paper demonstrated not being packed in the emergency department and being on antiplatelet medications were significant predictors of representation within 10 days [1]. Our study shows patients to be on an anticoagulant and having certain co-morbidities, such as AF, with epistaxis and potential rebleed rates. These factors could impact the chances of successfully managing epistaxis on outpatient bases.

Our study also revealed a reduced length of inpatient stay in the second loop of the audit compared to the first (p <0.05). Reduced length of stay within the hospital may have positive financial implications. This was demonstrated in a study by McCrossan et al. looking at safely discharging patients home with rapid rhinos in situ. From this study “National Institute for Health and Care Excellence (NICE) costings statement” showed a drop in cost/bed-day expenses by approximately £11000 due to reduced length of inpatient stay [9]. Therefore, the “Pack and Home” criteria may influence financial constraints within hospital trusts and may increase bed availability in surgical wards.

Strengths of this audit study include successfully analyzing study during the pandemic under difficult circumstances. A key limitation of this audit is the lack of generalizability of the results. Our study population consists mainly of an elderly population which can impact the risk of rebleeding and raise safety concerns with regard to appropriate outpatient management [10]. A univariate analysis was not performed in this study, therefore, we were unable to adjust for potential confounding, despite there being minimal difference in baseline characteristics between the loop 1 and loop 2 cohorts as demonstrated in Table 1.

In summary, our audit study demonstrates the possibility of outpatient management of patients with epistaxis with nasal packs in situ. Consideration must be taken towards clinical characteristics of patients that meet the “Pack and Home” criteria to ensure successful and safe compliance with the pathway.

## Conclusions

The "Pack and Home" criteria successfully identify patients who are suited to outpatient management pathway. Three patients on the "Pack and Home" pathway represented back to hospital within 48 hours of which only one rebled and was therefore deemed as unsuccessful management. This pathway could reduce inpatient admissions, have positive financial implications, and ultimately impact bed availability for surgical patients.

To ensure that we can continue outpatient management of epistaxis as routine, clinicians are advised to be fully aware of the "Pack and Home" criteria before allocating patients to this pathway. Patients on the outpatient pathway should also be adequately counseled about what to do if a rebleed occurs with safety net.
